# A Comparative Analysis of iPSC, MSC, and 293T-Derived Exosomes for Parkinson’s Disease Therapy

**DOI:** 10.3390/ijms27104212

**Published:** 2026-05-09

**Authors:** Kuan-Fei Chen, Syuan-Ling Lin, Chuan-Hao Kuo, Woei-Cherng Shyu, Long-Bin Jeng, Shih-Ping Liu

**Affiliations:** 1 Graduate Institute of Biomedical Science, China Medical University, Taichung 411, Taiwan; kfchen126@gmail.com; 2 Department of Neurology, China Medical University Hospital, China Medical University, Taichung 404, Taiwan; 3 Translational Medicine Research Center, China Medical University Hospital, Taichung 404, Taiwan; irislin3316@gmail.com (S.-L.L.); colin061450@gmail.com (C.-H.K.); shyu9423@gmail.com (W.-C.S.); 4 Cell Therapy Center, China Medical University Hospital, Taichung 404, Taiwan; 5 Organ Transplantation Center, China Medical University Hospital, Taichung 404, Taiwan; 6 Ph.D. Program for Aging, College of Medicine, China Medical University, Taichung 404, Taiwan; 7 Neuroscience and Brain Disease Center, College of Medicine, China Medical University, Taichung 411, Taiwan; 8 Office of Research and Development, Asia University, Taichung 413, Taiwan

**Keywords:** Parkinson’s disease, induced pluripotent stem cell, exosome

## Abstract

Parkinson’s disease (PD) lacks effective therapeutic methods. Exosomes are specialized vesicles that feature a double-layered lipid structure and are rich in proteins, miRNA, mRNA, growth factors, and other biomolecules. Their diverse components enable tissue repair and cell activation, making exosomes a promising candidate for therapeutic applications, including for PD. Exosomes are widely studied in cancer treatment and regenerative medicine. Since these vesicles retain the characteristics of their source cells, selecting the appropriate cell type is crucial. In this study, we compared exosomes derived from induced pluripotent stem cells (iPSCs), mesenchymal stem cells (MSCs), and 293T cells in terms of particle production, protein content, cellular uptake efficiency, and therapeutic effects on PD. The results showed that exosomes derived from iPSCs outperformed those from the other two cell types in all evaluated aspects, followed by MSC-derived exosomes, while 293T-derived exosomes were the least effective. This study provides valuable comparative data to inform the selection of source cells for exosome-based therapies in regenerative medicine.

## 1. Introduction

Parkinson’s disease (PD) is clinically characterized by reduced dopamine (DA) levels in the striatum [1], primarily due to the progressive degeneration of neuromelanin (NM)-containing dopaminergic neurons in the substantia nigra pars compacta (SNc) [2]. Available therapies to alleviate PD symptoms are limited by debilitating side effects and loss of efficacy over time, as they do not modify the progression of the disease [3]. 

Exosomes are small extracellular vesicles (30–150 nm in diameter) secreted by most cell types into bodily fluids, such as blood, urine, and saliva. These vesicles are formed within multivesicular bodies (MVBs) and released into the extracellular environment through the fusion of MVBs with the plasma membrane [4]. Exosomes play critical roles in intercellular communication by transporting bioactive molecules, including proteins, lipids, and nucleic acids (e.g., mRNA and microRNA), from donor to recipient cells [5]. The biological functions of exosomes are diverse and context-dependent. In physiological conditions, they are involved in processes such as immune modulation, tissue repair, and neuronal signaling [6]. In pathological states, exosomes contribute to the progression of diseases, including cancer, neurodegeneration, and infectious diseases. For example, cancer-derived exosomes can promote tumor growth and metastasis by altering the microenvironment and modulating immune responses [7]. Exosomes have gained significant interest as diagnostic and therapeutic tools. Due to their ability to carry disease-specific biomarkers, these vesicles are being explored as non-invasive liquid biopsy tools for early disease detection [8]. Moreover, their natural capacity for cargo delivery has inspired their use as drug delivery vehicles, with engineered exosomes being developed to target specific cells or tissues. Previous studies have also suggested that PD may be associated with post-transcriptional regulatory mechanisms, such as alternative splicing. Since exosomes carry RNA and proteins, they may also be involved in this pathway [9].

Exosome-producing cells, such as HEK-293T cells, mesenchymal stem cells (MSCs), and induced pluripotent stem cells (iPSCs), are instrumental in studying exosome communication and therapeutic applications. These cells produce exosomes that contain bioactive molecules, which influence recipient cell behavior [5].

HEK-293T cells, derived from human embryonic kidney cells, are commonly used in exosome research due to their high exosome yield and ease of genetic manipulation [10]. These exosomes are often engineered for therapeutic applications, including drug delivery and targeted gene therapy [11]. MSCs are a prominent source of therapeutic exosomes due to their immunomodulatory and regenerative properties. MSC-derived exosomes promote tissue repair by transferring proteins, mRNAs, and microRNAs that modulate inflammation, angiogenesis, and cell proliferation [12]. They are being investigated in regenerative medicine, particularly for the treatment of myocardial infarction and osteoarthritis. iPSCs, reprogrammed from somatic cells, produce exosomes with potential applications in regenerative therapies. iPSC-derived exosomes have been shown to promote neural repair and restore cardiac function in preclinical studies, largely due to their cargo of regenerative molecules and genetic material [13].

Exosomes have emerged as a promising tool for understanding, diagnosing, and treating PD. These extracellular vesicles facilitate intercellular communication and carry biomolecules that are critical in PD pathophysiology [14]. Exosomes derived from neural cells can reflect pathological processes occurring in the brain, making them valuable for biomarker discovery. For example, α-synuclein, a protein implicated in PD, is often enriched in exosomes isolated from the cerebrospinal fluid and blood of patients with PD. Exosome α-synuclein levels can serve as potential diagnostic biomarkers and aid in monitoring disease progression [15]. 

Therapeutically, exosomes have shown potential as drug delivery systems for PD. Engineered exosomes can cross the blood–brain barrier (BBB) and deliver neuroprotective agents, such as small interfering RNAs (siRNAs) or therapeutic proteins, directly to affected brain regions [16,17]. Exosomes derived from MSCs are particularly promising, as they possess anti-inflammatory and neuroprotective properties that may mitigate neurodegeneration in PD models [18]. Recent research also highlights engineered exosomes, such as those containing melanin, which modulate astrocytic pathways to reduce PD pathology [19]. Despite these advances, further research is needed to optimize exosome production, loading, and targeting for clinical application in PD.

The MPTP-induced PD mouse model is a widely used experimental tool for studying neurodegeneration in PD. MPTP, a neurotoxin, selectively destroys dopaminergic neurons in the SNc, mimicking the clinical and pathological features of PD [20]. The compound crosses the BBB and is metabolized into MPP+ in glial cells, which is then taken up by dopaminergic neurons through dopamine transporters, leading to mitochondrial dysfunction and oxidative stress-induced cell death [21]. This model is advantageous for its reproducibility, simplicity, and ability to induce symptoms like PD, including bradykinesia, rigidity, and tremors [22]. Furthermore, MPTP administration can be acute, subacute, or chronic, depending on the desired level of neurodegeneration and motor deficits [23]. Studies have demonstrated that the MPTP model is useful for assessing neuroprotective strategies, drug efficacy, and the role of neuroinflammation in PD pathology [24].

Since the origin of cells used for exosome production is critical, a thorough assessment of the selected cell types is essential. In this study, we compared exosomes derived from 293T cells, MSCs, and iPSCs by analyzing their particle production, protein content, cellular uptake efficiency, and therapeutic effects on PD, laying a foundation for future research and commercial applications.

## 2. Results

### 2.1. Characteristics of 293T-, MSC-, and iPSC-Derived Exosomes

**Figure 1:**
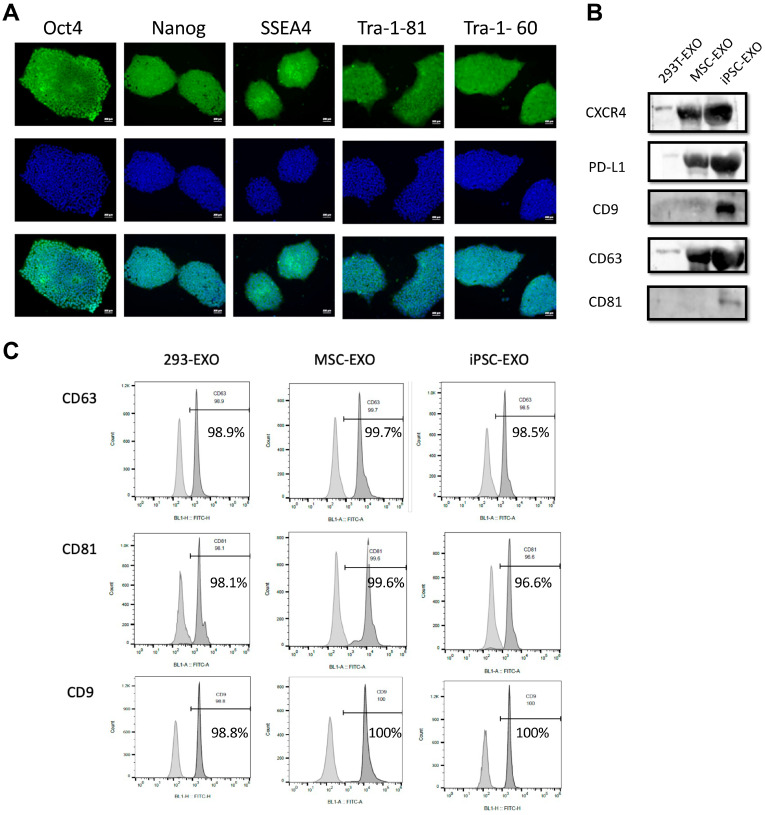

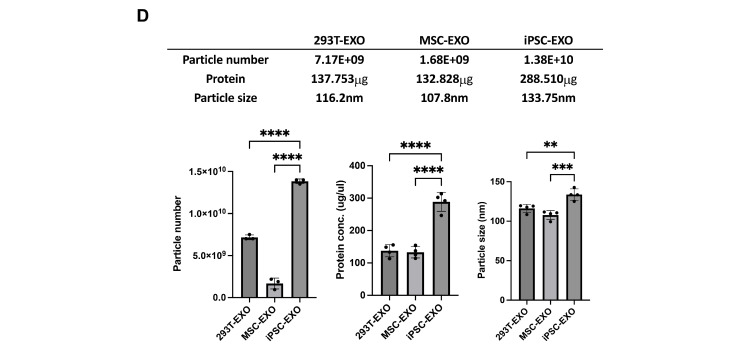
Characterization of 293T-, MSC-, and iPSC-derived exosomes. (**A**) Immunofluorescent staining of pluripotency markers OCT4, NANOG, SSEA4, TRA-1-81, and TRA-1-60 in iPSCs (red). Nuclei were stained with DPAI (blue). (**B**) Western blot and (**C**) flow cytometry of specific exosome surface markers CD63, CD9, and CD81, as well as the PD-L1 and CXCR4, in 293T-, MSC-, and iPSC-derived exosomes. (**D**) The particle numbers, protein concentrations, and particle size in 293T-, MSC-, and iPSC-derived exosomes. Difference between groups was evaluated by one-way analysis of variance with Newman–Keuls Multiple Comparison Test (** *p* < 0.01, *** *p* < 0.001, **** *p* < 0.0001).

The pluripotency of iPSCs was confirmed via immunocytochemistry to detect the expression of key pluripotency markers, including OCT4, Nanog, SSEA4, TRA-1-81, and TRA-1-60 (Figure 1A). Exosomes derived from 293T cells (293T-EXO), MSCs (MSC-EXO), and iPSCs (iPSC-EXO) were individually isolated from their respective conditioned media. Western blot analysis verified the presence of the exosome markers CD9, CD63, and CD81, confirming the exosome nature of the isolated iPSC-EXO (Figure 1B). Flow cytometry analysis further demonstrated that the expression of exosome markers CD63 and CD81 on the produced 293T-EXO, MSC-EXOs, and iPSC-EXOs was greater than 96.6% (Figure 1C). In addition, the expression levels of CXCR4 (homing factor) and PD-L1 (anti-inflammatory factor) were higher in iPSC-EXO than those in 293T-EXO and MSC-EXO. Nanoparticle tracking analysis (NTA) demonstrated that the particle size of 293T-EXO, MSC-EXO, and iPSC-EXO ranged from 119 to 178 nm (Figure 1D). The yield of iPSC-EXOs (including particle number and protein) was more than ten times higher than that of 293T-EXO and MSC-EXO (Figure 1D). These data suggest that iPSC-EXO have greater potential than 293T-EXOs and MSC-EXO for future applications.

### 2.2. Comparison of 293T-EXO, MSC-EXO, and iPSC-EXO Uptake Ability in SH-SY5Y

**Figure 2:**
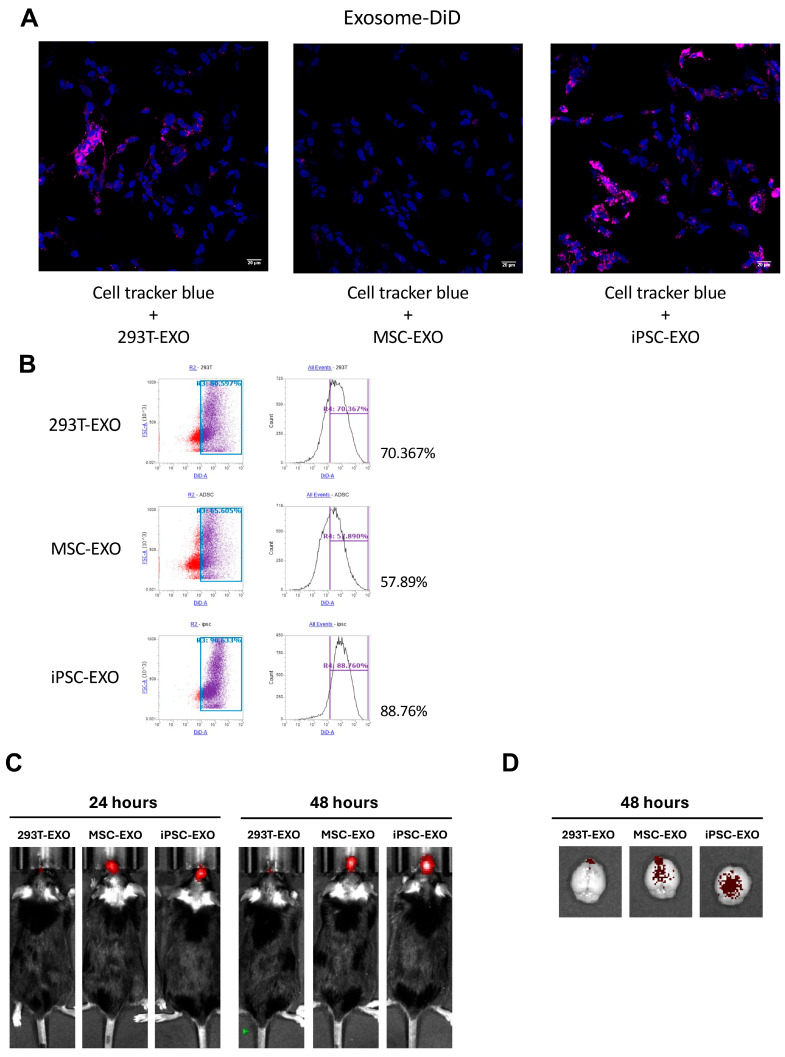
Characterization of the uptake ability and biodistribution of DiD-293T-, DiD-MSC-, and DiD-iPSC-derived exosomes. (**A**) SH-SY5Y cells were treated with DiD-293T, DiD-MSC, and DiD-iPSC-derived exosomes to observe uptake ability. (**B**) Intracellular uptake of DiD-labeled exosomes derived from 293T cells, MSCs, and iPSCs by SH-SY5Y cells was quantified using flow cytometry, based on the measurement of fluorescence intensity. (**C**) The fluorescence intensity at 24 and 48 h after exosome treatment, using an IVIS system. (**D**) The brain fluorescence intensity of exosome treatment at 48 h after exosome treatment.

The cellular uptake of exosomes is critical to determine their effectiveness for therapeutic purposes. To evaluate this, SH-SY5Y cells were treated with 293T-EXO, MSC-EXO, and iPSC-EXO, and their endocytosis ability was assessed using immunofluorescence staining and flow cytometry. Immunofluorescence staining revealed that the number of iPSC-EXOs entering the cells was significantly higher compared to 293T-EXO and MSC-EXO (Figure 2A). Flow cytometry analysis confirmed these findings: 88.8% of cells internalized iPSC-EXO, whereas only 70.4% and 57.9% of cells internalized 293T-EXO and MSC-EXO, respectively (Figure 2B). We also conducted an in vivo biodistribution study. Mice were administered MPTP, and on day 7, DiD-labeled 293T-EXO, MSC-EXO, or iPSC-EXO were injected. Exosome distribution was monitored using IVIS imaging at 24 and 48 h post-injection. At 48 h, the mice were sacrificed, and exosome distribution in the brain was examined. The results showed that the iPSC-EXO-treated group exhibited the highest accumulation and the widest distribution of exosomes in the brain (Figure 2C,D).

### 2.3. iPSC-EXO Significantly Attenuated the MPP^+^-Induced SH-SY5Y Cells Apoptosis In Vitro

**Figure 3:**
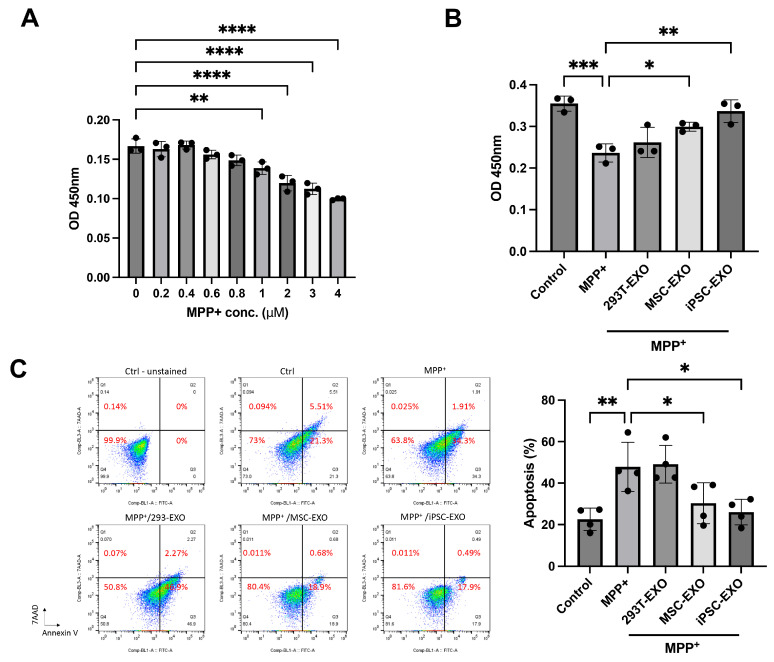
Demonstration of the MPP^+^ induced SH-SY5Y cells apoptosis after 293T-, MSC-, and iPSC-derived exosome treatment. (**A**) The CCK8 assay of Sh-SY5Y cells treated with various concentrations of MPP^+^. (**B**) The CCK8 assay was used to assess the cell viability of SH-SY5Y cells treated with 2 μM MPP^+^ combined with saline, 293T-EXO, MSC-EXO, or iPSC-EXO for 24 h. (**C**) Annexin V-FITC/7-AAD double staining was used to detect cell apoptosis induced by MPP^+^ in SH-SY5Y cells treated with saline, 293T-EXO, MSC-EXO, or iPSC-EXO for 24 h. Difference between groups was evaluated by one-way analysis of variance with Newman–Keuls Multiple Comparison Test (* *p* < 0.05; ** *p* < 0.01, *** *p* < 0.001, **** *p* < 0.0001).

To evaluate whether 293T-EXO, MSC-EXO, and iPSC-EXO could mitigate MPP^+^-induced cell death, the optimal concentration and treatment duration of MPP^+^ were first determined. SH-SY5Y cells were exposed to varying concentrations of MPP^+^ (0, 0.2, 0.4, 0.6, 0.8, 1, 2, 3, and 4 μM) for 24 h. Cell viability was measured using the Cell Counting Kit-8 (CCK-8) assay and expressed as a percentage relative to untreated controls. The results showed that MPP^+^ treatment significantly induced cell death in SH-SY5Y cells in a concentration-dependent manner over the 24 h period (Figure 3A). Based on these results, 2 μM was selected as the optimal MPP^+^ treatment dose for subsequent experiments. The CCK-8 assay revealed a significantly greater reduction in MPP^+^-induced cell death following treatment with iPSC-EXO compared to pretreatment with 293T-EXO and MSC-EXO, as well as the control group (Figure 3B). Consistently, treatment with iPSC-EXO significantly reduced apoptosis following MPP^+^ exposure, compared to treatment with 293T-EXO, MSC-EXO, and the untreated control group, as demonstrated by Annexin V-fluorescein isothiocyanate (FITC)/7-aminoactinomycin D (7-AAD) double staining (Figure 3C).

### 2.4. RNA Sequencing 

**Figure 4:**
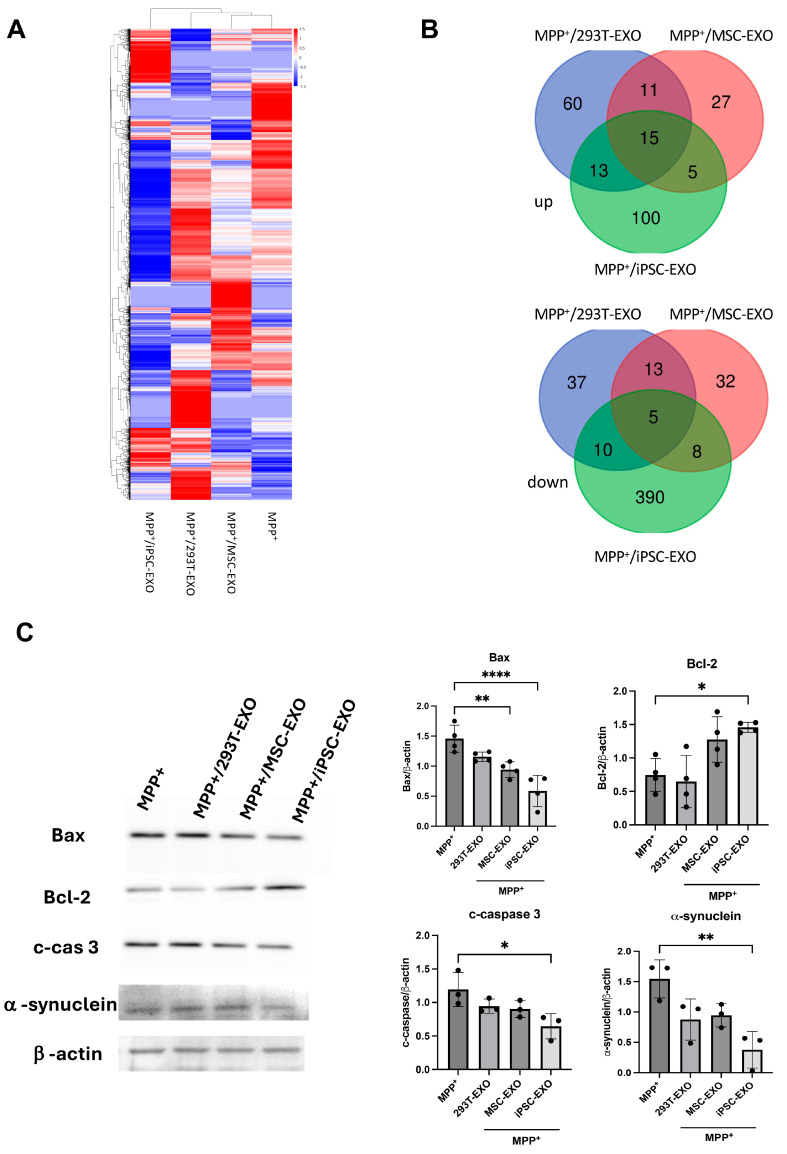
Identification of differentially expressed genes in SH-SY5Y cells treated with MPP^+^ combined with saline, 293T-EXO, MSC-EXO, or iPSC-EXO. (**A**) The clustering heatmaps show gene expression profiles in the four groups. (**B**) Compared to the MPP^+^ group, the MPP^+^ + iPSC-EXO group shows a twofold or greater change in gene expression, with 100 genes upregulated and 390 genes downregulated. (**C**) Western blot of proteins (e.g., Bax, Bcl-2, cleavage caspase-3, and α-synuclein) involved in the MPP^+^-induced apoptosis of SH-SY5Y cells. Difference between groups was evaluated by one-way analysis of variance with Newman–Keuls Multiple Comparison Test (* *p* < 0.05, ** *p* < 0.01, **** *p* < 0.0001).

To investigate the underlying mechanism by which exosomes influence SH-SY5Y cells treated with MPP^+^, we conducted RNA-based next-generation sequencing to examine transcriptomic changes in SH-SY5Y cells exposed to MPP^+^ in combination with PBS, 293T-EXO, MSC-EXO, or iPSC-EXO. Multivariate clustering heatmap analysis revealed that the MPP^+^ group first clustered with the MPP^+^ + 293T-EXO group, followed by the MPP^+^ + MSC-EXO group. Notably, the gene expression profile of the MPP^+^ + iPSC-EXO group was the most distinct from the MPP^+^-only group (Figure 4A), suggesting that treatment with iPSC-EXO resulted in the most pronounced transcriptomic alterations. To further investigate the functional significance of these changes, gene set enrichment analysis (GSEA) was performed to identify overlapping genes across the four treatment groups. The analysis showed that iPSC-EXO treatment led to the upregulation of approximately 100 genes and downregulation of 390 genes, in contrast to the 293T-EXO and MSC-EXO groups, which exhibited minimal gene expression changes (Figure 4B). KEGG pathway analysis further demonstrated that the apoptosis pathway was significantly affected in the iPSC-EXO-treated group, with a high proportion of genes exhibiting altered expression, while no such changes were detected in the 293T-EXO and MSC-EXO groups (Table 1). Additionally, in SH-SY5Y cells treated with iPSC-EXO for 24 h combined with MPP^+^ administration, the expression levels of the proapoptotic proteins Bax and cleaved caspase-3 were markedly decreased, while the levels of the antiapoptotic protein Bcl-2 were significantly elevated, compared to those observed with 293T-EXO or MSC-EXO treatment and the control (Figure 4C). In addition, the expression levels of α-synuclein were significantly reduced in the iPSC-EXO-treated group compared to the other three groups (Figure 4C). Collectively, these results suggested that iPSC-EXO exhibit neuroprotective effects against MPP^+^-induced cytotoxicity. 

### 2.5. iPSC-EXO Treatment Improved Behavioral Recovery After PD Induction

**Figure 5:**
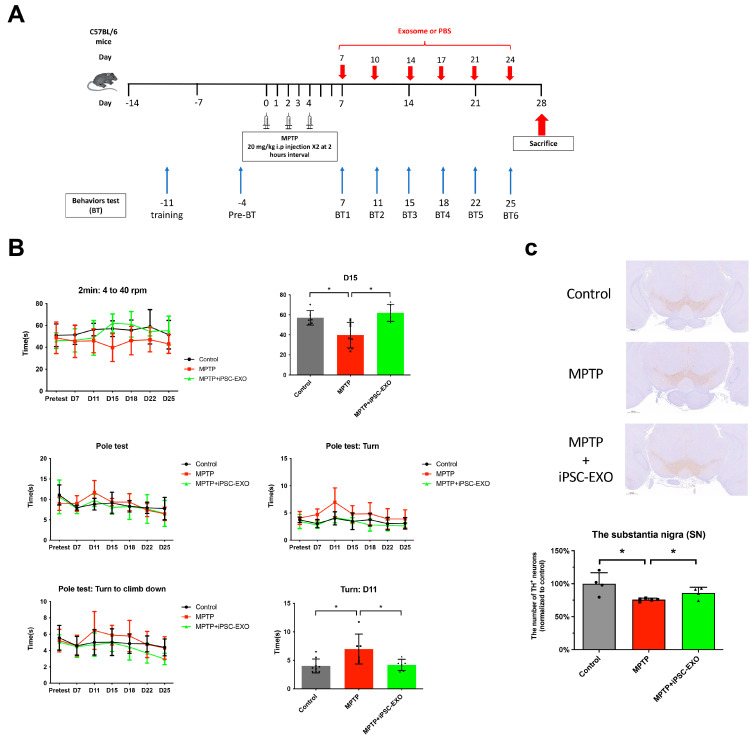
Evaluation of the therapeutic effects of iPSC-EXO in behavioural assays. (**A**) Schematic diagram outlining the MPTP treatment regimen and the timeline for behavioural testing. (**B**) Therapeutic efficacy of iPSC-EXO was evaluated using the pole test and rotarod performance analysis. (**C**) Assessment of tyrosine hydroxylase (TH)-positive neurons in the brains of PD model mice. Representative images show TH-positive cells in the substantia nigra (SN) region. Brain tissues were collected from C57BL/6 wild-type (WT) mice and mice that underwent behavioural testing. Difference between groups was evaluated by one-way analysis of variance with Newman–Keuls Multiple Comparison Test (* *p* < 0.05).

We modified a previously established MPTP-induced mouse model of PD to evaluate the therapeutic effects of exosomes. The neuronal behaviors of B6 mice, including performance on the rotarod and pole tests, were assessed on day -1 after two separate training sessions. On days 0, 2, and 4, male B6 mice were intraperitoneally injected with MPTP at a dose of 20 mg per 10 kg body weight, administered twice daily at 2 h intervals. Exosome treatment was delivered via intranasal administration twice a week for three weeks, starting on day 7. The recovery of behavioral function was evaluated on days 7, 11, 15, 18, 22, and 25 (Figure 5A). The rotarod data demonstrated that treatment with iPSC-EXO significantly improved the recovery of balancing ability in PD mice by day 15. While PD mice treated with saline showed a continued decline in the time spent on the rotarod, those treated with iPSC-EXO exhibited a significant improvement compared to the MPTP-only group (Figure 5B). In the pole test, three parameters were evaluated: total time, turn time, and turn-to-climb-down time. While all three metrics indicated the efficacy of iPSC-EXO, the most pronounced improvement was observed in the turn time. On day 11, the turn time was significantly reduced in the iPSC-EXO-treated group compared to the MPTP-only group, indicating that iPSC-EXO effectively improved the balance ability of PD mice (Figure 5B).

### 2.6. Changes in the Number of TH-Positive Cells After iPSC-EXO Treatment

Our behavioral study demonstrated that iPSC-EXO treatment significantly improved post-PD recovery. To further confirm these findings, mice from each group were collected on day 28, and sections of the substantia nigra (SN) region were stained for the dopaminergic cell marker tyrosine hydroxylase (TH) using immunohistochemistry (IHC). Cell count analysis revealed that the average number of TH-expressing cells decreased by approximately 50% following PD induction (Figure 5C). However, the number of TH-expressing cells increased in PD mice treated with iPSC-EXOs, aligning with the results of our behavioral study. These findings suggested that iPSC-EXOs exert superior therapeutic effects, promoting both behavioral recovery and dopaminergic neuron preservation in PD mice.

## 3. Discussion

PD is a progressive neurodegenerative disorder characterized by the loss of dopaminergic neurons in the SN and the presence of Lewy bodies. Exosomes derived from various cell types, including iPSCs, MSCs, and HEK-293T cells, are being increasingly investigated as potential therapeutic or diagnostic tools for PD. Among these, iPSC-derived exosomes offer unique advantages in regenerative medicine, while MSC- and HEK-293T-derived exosomes contribute to neuroprotection, drug delivery, and mechanistic understanding.

iPSC-derived exosomes have shown promising therapeutic potential in PD models due to their contents of neurotrophic factors, miRNAs, and anti-inflammatory molecules. iPSCs can be differentiated into neural progenitor cells or dopaminergic neurons, allowing for the production of exosomes that more closely mimic the neural microenvironment. Published research on iPSC-derived exosomes for PD treatment remains scarce. However, some studies have shown that iPSC-NSCs-derived exosomes let-7b-5p improves motor function after spinal cord injury by modulating microglial/macrophage pyroptosis [25]. Although let-7b-5p was not specifically examined in this study, it is possible that it may exert similar mechanisms in promoting neural repair. Moreover, because iPSCs can be generated from patient-specific cells, they offer the potential for personalized exosome-based therapies that minimize immune rejection and are tailored to individual molecular profiles.

In contrast, MSC-derived exosomes are already well-studied for their neuroprotective and anti-inflammatory effects in PD models. MSCs from various sources—such as bone marrow, adipose tissue, and umbilical cord—secrete exosomes rich in growth factors, cytokines, and regulatory RNAs that can cross the BBB and mitigate neuronal damage [26]. For instance, MSC-derived exosomes have been shown to attenuate dopaminergic neuronal loss, reduce glial activation, and restore motor function in animal models of PD [27]. One of the main advantages of MSC exosomes lies in their immunomodulatory capabilities, which help create a neuroprotective environment and reduce chronic inflammation associated with PD pathology [26,27]. However, donor variability and source heterogeneity can lead to inconsistent therapeutic effects, a challenge that iPSCs may help overcome with standardized differentiation protocols [25,28]. In this study, we confirmed that, in terms of therapeutic efficacy, iPS-derived exosomes are indeed superior to those derived from MSCs and 293T cells.

HEK-293T cell-derived exosomes, while lacking intrinsic regenerative or neuroprotective properties, have emerged as effective vehicles for exosome engineering in PD research. Their ease of culture and high transfection efficiency make them suitable for packaging therapeutic cargos such as siRNAs, CRISPR/Cas9 components, or small molecules targeting α-synuclein aggregation, a hallmark of PD pathology [29]. Although HEK-293T-derived exosomes are not inherently therapeutic, they are instrumental in proof-of-concept studies and exosomes drug delivery strategies, particularly in targeting neuronal cells through surface modification or ligand display.

Overall, while MSC exosomes have established themselves as a safe and effective neuroprotective agent, iPSC-derived exosomes offer a more customizable and potentially more disease-specific platform for treating PD. Their capacity for patient-specific customization and neurogenic potential positions them as a promising next-generation therapy. Meanwhile, HEK-293T-derived exosomes continue to serve an essential role in exosome-based engineering and therapeutic delivery. In addition, previous studies have shown that brain changes associated with deviant peer environments closely correspond to the distribution patterns of neurotransmitters such as dopamine and serotonin. It may be worthwhile to investigate this from the perspective of exosomes and explore their potential therapeutic applications [30]. Future work should focus on comparative in vivo studies, scalable production methods, and the development of targeted delivery systems to translate these findings into clinical applications for PD.

## 4. Materials and Methods

### 4.1. Cell Culture 

To maintain Lenti-X™ 293T cells (Takara Bio Inc, Kusatsu, Shiga, Japan) in optimal condition, Dulbecco’s Modified Eagle’s Medium (DMEM) supplemented with 10% fetal bovine serum (FBS) was used. The growth medium was replaced every 2–3 days.

Human telomerase reverse transcriptase-immortalized adipose tissue-derived MSCs (hTERT-ADSCs or ASC52telo; Cat# SCRC-4000) were procured from the American Type Culture Collection (ATCC). These cells were cultured in MSC NutriStem XF Medium and Supplement Mix (Sartorius, Göttingen, Germany). 

Human healthy iPSCs (IBMS-iPSC-02-07_feeder-free) were obtained from the Bioresource Collection and Research Center (BCRC). The iPSCs were subcultured for fewer than 10 passages post-resuscitation. iPSCs were cultured in StemFit medium (Cat# SF041-001, Ajinomoto, Tokyo, Japan), and accutase (Cat# SCR005, Millipore, Burlington, MA, USA) was used for subculturing. After detachment from the culture dish, the ROCK inhibitor Y-27632 (Cat# 10005583, Cayman Chemical, Ann Arbor, MI, USA) was added to the culture medium to prevent cell death and promote attachment. The culture medium for iPSCs was refreshed daily to ensure a stable culture environment.

Human SH-SY5Y cells were cultured in DMEM/F12 (1:1, *v*/*v*) (Cat# 11330032, Gibco, Grand Island, NY, USA) supplemented with 10% FBS (Cat# SH30084.03, Cytiva, Marlborough, MA, USA) and 1% penicillin–streptomycin (Cat# 15140122, Gibco, Grand Island, NY, USA). These cells were maintained in a humidified atmosphere with 5% CO_2_ at 37 °C.

### 4.2. Validation of the iPSCs 

Immunofluorescent staining was carried out following the protocol outlined in a previous study [31]. The following antibodies were used for staining: anti-Oct4 (Cat# GTX101497, GeneTex, Irvine, CA, USA), anti-Nanog (Cat# GTX100863, GeneTex, Irvine, CA, USA), anti-stage-specific embryonic antigen 4 (Cat# part893871, R&D systems, Minneapolis, MN, USA), anti-TRA-1-81 (Cat# MAB4381, Millipore, Burlington, MA, USA), and anti-TRA-1-60 (Cat# MAB4360, Millipore, Burlington, MA, USA).

### 4.3. Purification of 293T-, MSC-, and iPSC-Derived Exosomes

Cells were seeded at a density of 1 × 10^6^ in T75 flasks and cultured for two days. The medium was then replaced, and the conditioned medium collected over the subsequent two days was used for exosome isolation. To isolate exosomes, the culture supernatants were initially centrifuged at 500× *g* for 10 min, followed by 3000× *g* for 20 min to remove cells and debris. The resulting clarified supernatants were concentrated using Amicon Ultra-15 Centrifugal Filter Units with a 10 kDa molecular weight cutoff (Cat# UFC901024, Millipore, Burlington, MA, USA) and further centrifuged at 5000× *g* for 1 h at 4 °C. The concentrated medium was then filtered through 0.22-μm pore size filters. Subsequently, samples were incubated overnight at 4 °C with Total Exosome Isolation Reagent (Cat# 4478359, Thermo Fisher Scientific, Waltham, MA, USA). Exosomes were pelleted through centrifugation at 10,000× *g* for 1 h at 4 °C. The isolated exosomes were either used immediately or resuspended in sterile 1× PBS and stored at −80 °C for future use.

### 4.4. Nanoparticle Tracking Analysis

Exosomes were resuspended in sterile 1× PBS to a final volume of 1 mL. Their size and concentration were determined using the ZetaView MONO NTA system (Particle Metrix, Inning am Ammersee, Germany), which features high-speed video capture and particle-tracking capabilities via ZetaView Software version 8.04.02 SP2.

### 4.5. Western Blot Analysis

Whole-cell lysates or exosome proteins were separated using 8–10% sodium dodecyl sulfate-polyacrylamide gel electrophoresis (SDS-PAGE) and subsequently transferred onto nitrocellulose membranes. The membranes were blocked with 5% nonfat dry milk at 25 °C for 1 h and incubated overnight at 4 °C with the appropriate primary antibodies at supplier-recommended dilutions. This was followed by incubation with horseradish peroxidase-conjugated secondary antibodies at room temperature for 1 h. The membranes were developed using enhanced chemiluminescence (ECL) detection reagents (Thermo Fisher Scientific, Waltham, MA, USA). Exosome markers included CD63 (clone: Ts63, Thermo Fisher Scientific, Waltham, MA, USA), CD9 (clone: ARC0330, ABclonal, Woburn, MA, USA), and CD81 (clone: ARC0615, ABclonal, Woburn, MA, USA). Other proteins were detected using antibodies against PD-L1 (Cat# GTX635975, GeneTex, Irvine, CA, USA), CXCR4 (Cat# GTX638646, GeneTex, Irvine, CA, USA), Bcl-2 (Cat# GTX100064, GeneTex, Irvine, CA, USA), Bax (Cat# GTX109683, GeneTex, Irvine, CA, USA), and cleaved caspase 3 (Cat# AB32351, Abcam, Cambridge, UK). Beta-actin (Cat# GTX629630, GeneTex, Irvine, CA, USA) was used as the loading control.

### 4.6. Flow Cytometry of Exosome CD63, CD9, and CD81

Exosomes were resuspended in sterile 1× PBS and incubated overnight at 4 °C with streptavidin-coated magnetic beads from the Exo-Flow Capture Kit (Cat# EXOFLOW150A-1, System Biosciences, Palo Alto, CA, USA). The beads were pre-coupled with biotinylated tetraspanin antibodies (CD63, CD9, and CD81) provided in the kit, along with biotin-conjugated anti-PD-L1 (clone: 29E.2A3) and anti-CXCR4 (clone: 12G5) antibodies (BioLegend, San Diego, CA, USA). Following three washes to remove unbound exosomes, the captured exosomes were stained with Exo-FITC for 2 h at 4 °C in preparation for flow cytometric analysis. Data acquisition was performed using an Attune NxT Flow Cytometer, and gate settings as well as data analysis were conducted using FlowJo software (version 7.6). 

### 4.7. Exosome Labeling Using Lipophilic Membrane Dyes and Uptake by SH-SY5Y Cells In Vitro

Exosomes were labeled with 3.33 μg/mL DiD deep red dye (Thermo Fisher Scientific, Waltham, MA, USA) for 30 min at room temperature. The labeled exosomes were re-pelleted using Total Exosome Isolation reagent (1:1) overnight at 4 °C. Following centrifugation at 10,000× *g* for 1 h at 4 °C, the DiD-labeled exosomes were resuspended in sterile 1× PBS. Subsequently, the DiD-labeled exosomes were incubated with SH-SY5Y cells for 24 h at 37 °C. Images of exosome uptake were captured using an SP2 Confocal Spectral Microscope. Cell nucleus was stained using DAPI.

### 4.8. In Vitro Cytotoxicity Assay

SH-SY5Y cells were seeded in a 24-well plate and incubated overnight. The cells were then treated with varying concentrations of hydrogen peroxide (MPP^+^; 0, 0.2, 0.4, 0.6, 0.8, 1, 2, 3, and 4 μM) for 24 h in a humidified incubator at 37 °C with 5% CO_2_. Based on the results, 2 μM MPP^+^ was chosen to evaluate the neuroprotective effects of exosomes against MPP^+^-induced cell death. To test this, SH-SY5Y cells were treated with Exosome (1 × 10^9^ particle) and 2 μM MPP^+^ for 48 h. Cell viability was assessed using the CCK-8 assay, following the manufacturer’s protocol. Briefly, 10 μL of CCK-8 solution was added to each well containing 100 μL of culture medium, and the plate was incubated at 37 °C for 2 h. Absorbance was measured at 450 nm using a microplate reader.

### 4.9. Annexin V Fluorescence Assay

The apoptotic effect on SH-SY5Y cells was analyzed using an Annexin V fluorescence kit (BD Biosciences, Franklin Lakes, NJ, USA) following the manufacturer’s instructions. Briefly, cells were seeded in a six-well plate and incubated for 24 h. The seeded cells were divided into four groups: control, 293T-EXO, MSC-EXO, and iPSC-EXO (20 μg each). These groups were treated for 48 h with exosomes and 2 μM MPP^+^. After treatment, the cells were harvested and collected as pellets. The pellets were resuspended in 400 μL of binding buffer and stained with 5 μL of FITC-Annexin V and 5 μL of 7-AAD, as per the kit’s protocol. The stained cells were analyzed using an Attune NxT Flow Cytometer, and the results were processed using FlowJo software v.7.6.

### 4.10. RNA Sequencing

SH-SY5Y cells treated with MPP^+^ combined with PBS, 293T-EXO, MSC-EXO, and iPSC-EXO were collected after 24 h, followed by total RNA extraction using the PureLink RNA Mini Kit (Thermo Fisher Scientific, Waltham, MA, USA), according to the manufacturer’s instructions. Total RNA lysates from the treated SH-SY5Y cells were used to construct RNA sequencing libraries. Total RNA quality and quantity were evaluated using NanoDrop spectrophotometer and Agilent Bioanalyzer 2100 with RIN determined by the RNA 6000 Nano assay.

RNA sequencing libraries were prepared using the SureSelect XT HS2 mRNA Library Preparation Kit (Agilent, Santa Clara, CA, USA) with 500–1000 ng of RNA as the input. Poly-A-tailed mRNA was enriched, and library quality was assessed using the Qubit dsDNA HS Assay and Agilent 4200 TapeStation. Sequencing on the Illumina NovaSeq X Plus system generated FASTQ files with over 20 million reads per sample.

Low-quality reads were trimmed with Trimmomatic v0.36, mapped using HISAT2 v2.2.1, and analyzed for gene expression with StringTie v2.1.7. Gene counts were normalized to TPM (Transcripts Per Million). Differential expression analysis was performed using DESeq, with annotations retrieved from Human GRCh38. Functional annotation and enrichment analysis were conducted using clusterProfiler v4.7.1. 

### 4.11. Animal Treatment 

C57BL/6 (B6) male mice (BioLASCO, Taipei City, Taiwan) were housed in individually ventilated cages at the Laboratory Animal Service Center. All animal treatments and experimental procedures were approved by the Institutional Animal Care and Use Committee of China Medical University (approval number: 2024-214-1). PD was induced in 8-week-old mice. 1-Methyl-4-phenyl-1,2,3,6-tetrahydropyridine hydrochloride (MPTP-HCl) (Sigma-Aldrich, St. Louis, MO, USA) was prepared as a stock solution in PBS at a concentration of 7 mg/mL. MPTP was administered via intraperitoneal injections at a dose of 20 mg/kg, with two injections given at 2 h intervals daily for five consecutive days. Mice treated with MPTP were randomized and blinded for each experimental group to ensure unbiased results.

### 4.12. Exosome Treatment

Exosomes were administered via intranasal delivery twice a week for three weeks, starting three days after the last MPTP treatment. Briefly, 1 × 10^9^ exosome particles suspended in 24 μL of PBS were administered intranasally. 

### 4.13. Neuronal Behaviors of Mice Before and After PD Induction

B6 mice underwent two training sessions on each apparatus prior to the experiment. Neuronal behaviors were assessed using two different devices: the pole test, used to monitor balancing ability, and the rotarod test, which is used to evaluate coordination. The animals’ coordination was measured as the duration they could remain stable on a rotating rod, with the speed increasing from 4 to 40 rpm over a 2 min period. Data from at least three independent experiments were collected for statistical analysis. The data collection period began on day 0 and continued until day 25, by which time MPTP-induced PD symptoms were no longer detectable in control mice. Following the behavioral assessment, the mice were sacrificed, and their brain tissues were collected. Additionally, brain samples from each group were gathered for pathological analysis.

### 4.14. Assessment of Dopaminergic Neuron Numbers

Following the behavioral study, frozen sections of brain tissue were prepared. The dopaminergic cell marker tyrosine hydroxylase (TH; Millipore, Burlington, MA, USA) was stained in sections of the SN. The number of TH-positive cells was quantified using ImageJ software version 1.54 (National Institutes of Health, Bethesda, MD, USA).

### 4.15. Statistical Analysis

Data from at least three independent experiments were analyzed for statistical significance using Prism software (version 5.01, San Diego, CA, USA) at a significance threshold of *p* < 0.05 (* *p* < 0.05, ** *p* < 0.01, *** *p* < 0.001). General Data Analysis: One-way analysis of variance (ANOVA), followed by the appropriate multiple comparisons test, was used to determine statistical significance. Results were expressed as the mean ± SD. Behavioral Data: Two-way ANOVA followed by a post hoc Bonferroni test was used for behavioral comparisons. Data were presented as the mean ± SEM. Variables with significant *p*-values in the univariate analysis were further analyzed using a Cox proportional hazards regression model for multivariate analysis. 

## 5. Conclusions

Among: the three sources, iPSC-derived exosomes consistently showed the highest efficacy across all the assessed criteria, with MSC-derived exosomes showing intermediate performance and 293T-derived exosomes demonstrating the lowest effectiveness.

## Figures and Tables

**Table 1:** Numbers of significantly deregulated genes with known biological functions classified according to KEGG and Babelomics databases.

	293T-EXO			MSC-EXO			iPSC-EXO		
Description	GeneRatio	BgRatio		GeneRatio	BgRatio		GeneRatio	BgRatio	
**Apoptosis**									
Apoptosis	0/137	0/8546	0%	0/137	0/8546	0%	4/137	137/8546	2.92%
**Signal transduction**									
Parkinson disease	3/271	271/8546	1.11%	4/274	271/8546	1.46%	16/271	271/8546	5.90%
ECM-receptor interaction	2/89	89/8546	2.04%	1/89	89/8546	1.12%	4/89	89/8546	4.49%
PPAR signaling pathway	0/76	0/8546	0%	0/76	0/8546	0%	3/76	76/8546	3.95%
cAMP signaling pathway	3/226	226/8546	1.33%	2/226	226/8546	0.88%	6/226	226/8546	2.65%
TGF-beta signaling pathway	1/108	108/8546	0.93%	0/108	0/8546	0%	3/108	108/8546	2.78%
MAPK signaling pathway	0/300	0/8546	0%	0/300	0/8546	0%	7/300	300/8546	2.33%
Wnt signaling pathway	2/174	174/8546	1.15%	0/174	0/8546	0%	3/174	174/8546	1.72%
JAK-STAT signaling pathway	2/168	168/8546	1.19%	0/174	0/8546	0%	1/168	168/8546	0.60%
Calcium signaling pathway	2/36	254/8546	0.01%	2/254	254/8546	0.79%	3/152	254/8546	0.01%
PI3K-Akt signaling pathway	1/362	362/8546	0.28%	2/362	362/8546	0.55%	3/362	362/8546	0.83%
**Cell proliferation**									
Cell cycle	0/158	0/8546	0%	0/158	0/8546	0%	6/158	158/8546	3.80%

## Data Availability

All relevant data supporting the findings of this study is available within this Manuscript. Any further question or request should be made to the corresponding authors.
